# Impaired bone formation in ovariectomized mice reduces implant integration as indicated by longitudinal *in vivo* micro-computed tomography

**DOI:** 10.1371/journal.pone.0184835

**Published:** 2017-09-14

**Authors:** Zihui Li, Gisela Kuhn, Michael Schirmer, Ralph Müller, Davide Ruffoni

**Affiliations:** 1 Institute for Biomechanics, ETH Zurich, Zurich, Switzerland; 2 pfm medical titanium gmbh, Nuremberg, Germany; 3 Mechanics of Biological and Bioinspired Materials Research Unit, Department of Aerospace and Mechanical Engineering, University of Liège, Liège, Belgium; Technische Universiteit Delft, NETHERLANDS

## Abstract

Although osteoporotic bone, with low bone mass and deteriorated bone architecture, provides a less favorable mechanical environment than healthy bone for implant fixation, there is no general agreement on the impact of osteoporosis on peri-implant bone (re)modeling, which is ultimately responsible for the long term stability of the bone-implant system. Here, we inserted an implant in a mouse model mimicking estrogen deficiency-induced bone loss and we monitored with longitudinal *in vivo* micro-computed tomography the spatio-temporal changes in bone (re)modeling and architecture, considering the separate contributions of trabecular, endocortical and periosteal surfaces. Specifically, 12 week-old C57BL/6J mice underwent OVX/SHM surgery; 9 weeks after we inserted special metal-ceramics implants into the 6^th^ caudal vertebra and we measured bone response with *in vivo* micro-CT weekly for the following 6 weeks. Our results indicated that ovariectomized mice showed a reduced ability to increase the thickness of the cortical shell close to the implant because of impaired peri-implant bone formation, especially at the periosteal surface. Moreover, we observed that healthy mice had a significantly higher loss of trabecular bone far from the implant than estrogen depleted animals. Such behavior suggests that, in healthy mice, the substantial increase in peri-implant bone formation which rapidly thickened the cortex to secure the implant may raise bone resorption elsewhere and, specifically, in the trabecular network of the same bone but far from the implant. Considering the already deteriorated bone structure of estrogen depleted mice, further bone loss seemed to be hindered. The obtained knowledge on the dynamic response of diseased bone following implant insertion should provide useful guidelines to develop advanced treatments for osteoporotic fracture fixation based on local and selective manipulation of bone turnover in the peri-implant region.

## Introduction

The remarkable load-bearing ability of healthy bone is based on a constant renewal of bone tissue as well as on a continuous adaptation of bone architecture to changes in the mechanical demands. The biological process responsible for bone maintenance and adaptation comprises both the removal of aged or mechanically redundant bone and the formation of new bone. Resorption and formation can either be spatially coupled with formation following resorption at the same site (a situation traditionally referred to as bone remodeling) or occurring at different locations (even far apart) and therefore allowing for growth and changes in bone architecture (a process named bone modeling). The general term used to describe all processes involving bone formation and resorption with no distinction between spatially coupled or un-coupled events is bone (re)modeling [1].

Impairments in the (re)modeling process such as increase in bone turnover together with an imbalance between formed and resorbed bone can lead to a rapid loss of bone mass [2] accompanied by a deterioration of bone architecture and material properties [3, 4], causing a generalized weakening of the bone [5]. This is a typical scenario occurring in osteoporotic individuals, which increases the likelihood for a bone fracture to happen under small loading forces (referred to as fragility fracture) [6]. Such fractures may need to be stabilized with orthopedic devices and the impact of osteoporosis on fracture fixation and implant anchorage is still rather controversial, mainly because of the lack of strong clinical evidence correlating the osteoporotic condition with implantation outcome [7]. Nevertheless, numerous biomechanical experiments suggest that implant anchorage in osteoporotic bone likely will be reduced [8–11]. Firstly, a peri-implant bone bed of low quality (typical in osteoporotic patients) presents less bone surface to anchor the implant as well as a weaker bone structure to receive the loads transmitted via the implant, therefore reducing the so-called initial (or primary) implant stability [8, 12, 13]. A second critical aspect for implant anchorage in osteoporotic individuals is the bone regeneration process taking place after implant insertion. This is a highly complex and dynamic activity which has several features in common with bone healing [14]: an initial inflammatory phase is usually followed by rapid bone formation, typically occurring directly on the implant surface to secure the implant [15], as well as by elevated bone resorption, especially targeted to remove peri-implant bone damaged caused by the insertion process [16]. After the acute healing response, bone (re)modeling continues within the peri-implant region and this process is ultimately responsible for the long-term (or secondary) stability of the bone-implant system. In osteoporotic subjects, bone (re)modeling can be disturbed: high turnover osteoporosis, for instance, is characterized by increased rates of bone resorption and formation (i.e. high bone remodeling), not only during the peri-menopausal period but also several years later [17]. Furthermore, bone healing, which combines traditional modeling and remodeling of bone, seems to be partially delayed in osteoporotic patients [18], although the precise reasons are still not well understood [19].

Implant placement modifies bone (re)modeling: recent studies using longitudinal *in vivo* micro-computed tomography (micro-CT) to monitor the spatio-temporal changes in peri-implant bone (re)modeling have demonstrated that bone responds to the presence of the implant essentially by increasing the rate of bone formation as well as of bone resorption in a time and location specific manner [20, 21]. Whether the ability of healthy bone to respond to implantation by rapidly adapting bone formation and resorption would be jeopardized in osteoporotic subjects is still an open question. This is highly relevant in the clinical setting for improving therapeutic modalities but cannot be assessed in a systematic way in human patients; thus, animal models are the preferred pre-clinical research approach. However, current results on peri-implant bone (re)modeling in ovariectomized (OVX) animals (which are used as surrogates of osteoporotic patients) are quite controversial. Some authors have found bone formation around implants to be significantly less in OVX animals [22–24] while others have reported no significant differences [25–28] or differences occurring only in the early stages of osseointegration [29]. In all the above mentioned studies bone (re)modeling has been measured with dynamic histomorphometry. This technique provides valuable information especially on the different tissues present in the peri-implant bone [14], but it has also recognized limitations when used to assess bone (re)modeling, the most serious being the impossibility of a proper characterization of bone resorption, the fact that it provides only two-dimensional information based on slices of the bone and therefore relative to a limited region, and its cross-sectional nature, which does not allow following the very same animal over time, therefore suffering from a higher variability [21, 30, 31].

In the present study, we investigated the time course of bone formation and resorption as well as the corresponding architectural modifications following the insertion of special metal-ceramics implants [20] into the 6^th^ caudal vertebra of mice. The animals at 12 weeks of age underwent OVX/SHM surgery; 9 weeks after ovaries removal we performed implantation and we monitored bone response for the following 6 weeks using longitudinal *in vivo* micro-CT imaging. We hypothesized that OVX animals would exhibit an impaired bone (re)modeling process with peri-implant bone formation and resorption being jeopardized by estrogen removal. We further hypothesized that such modifications would have a negative impact on peri-implant bone structure with a decreased ability to augment bone mass around the implant.

## Methods

### Animal experiment

**Fig 1:**
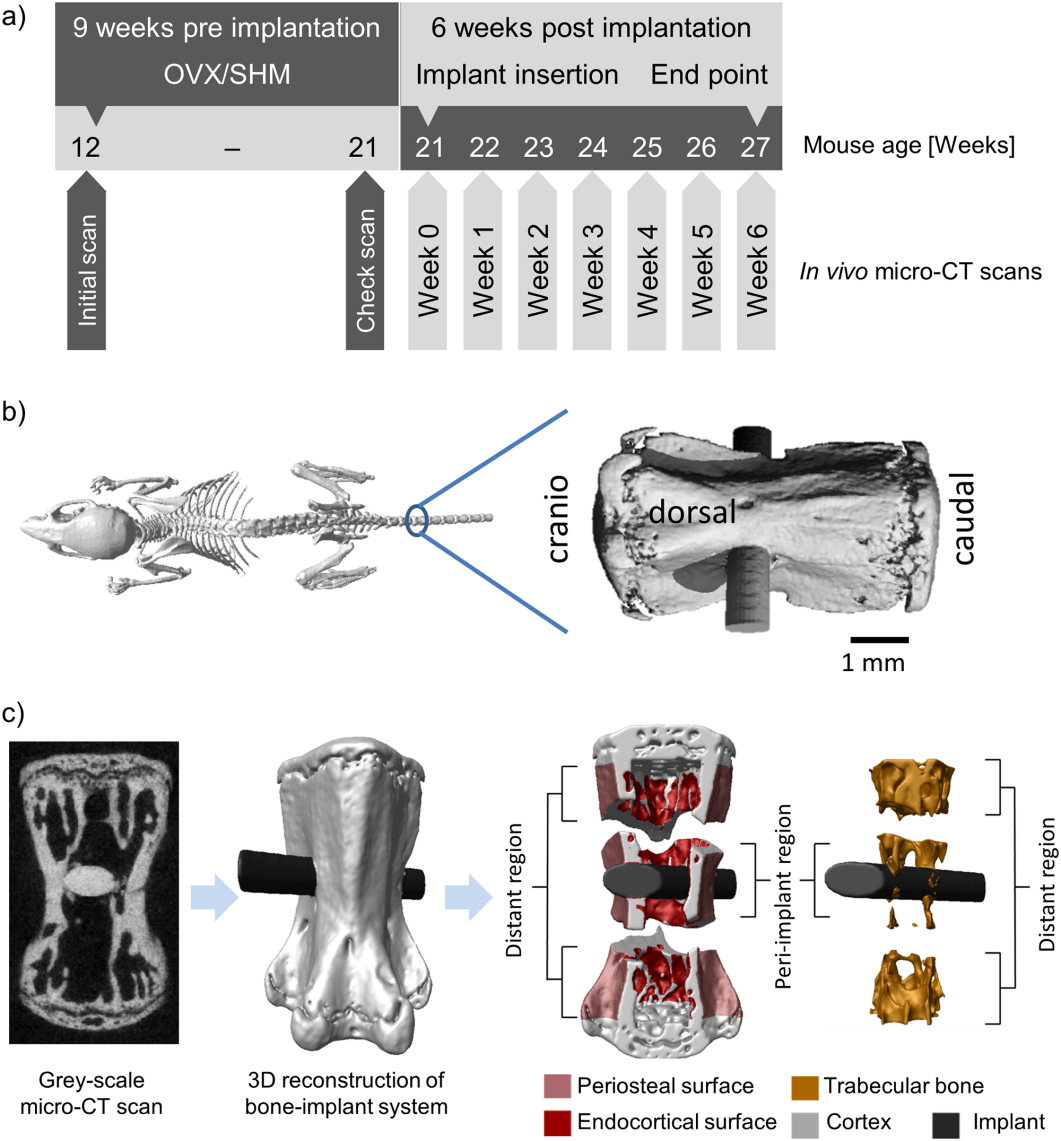
a) Timeline of the study: the mice were ovariectomized (OVX) at the age of 12 weeks and an initial scan was taken; 9 weeks after the surgery a check scan was done to confirm the bone loss; implant was inserted into the vertebra at the age of 21 weeks and a scan was performed right after implantation, followed by weekly scans for 6 weeks. The check scan and the scan at week0 were acquired one day apart. b) Schematic illustration of the implanted vertebra: the implant was inserted into CV6 along the left right axis. Image modified with permission from [1]. c) Image processing steps: the original grey-scale micro-CT scan (left) was segmented from the background and the three-dimensional reconstruction of the bone (light grey) with the implant (black) was produced (middle); different regions close to and far from the implant where bone (re)modeling was monitored were defined including periosteal and endocortical surfaces as well as trabecular bone (right).

For the animal experiment, described according to the ARRIVE Guidelines (S1 File), 12-week old female C57BL/6J mice (JANVIER, Saint Berthevin Cedex, France) were used. The mice were ovariectomized bilaterally (OVX group, n = 9) to provoke bone loss and structural deterioration. The sham group (SHM, n = 8) underwent surgery but the ovaries were not removed. We monitored body weight before and after OVX/SHM surgery for 3 days as an indicator of animal health, with the criterion that a weight loss larger than 10% would require intervention and higher than 15% euthanasia; however, both scenarios never happened as animals showed a maximum weight loss of 5% after surgery which was promptly recovered. After a recovery period of 9 weeks which allowed, in genetically identical mice of similar age, estrogen-deficient bone loss to reach a plateau [32], special needle-shape implants (Composite Metal Technology Limited, Hampshire, United Kingdom) were inserted into the sixth caudal vertebra (CV6) of both OVX and SHM animals. Implants and implantation procedure were already described in detail elsewhere [20]. In short, the implantation site was located with fluoroscopic imaging and small metal-ceramic implants (diameter of 0.5 mm and length of 1.5 mm) with a sharp tip were inserted using a well-established pinning procedure [33]. Implants were placed into CV6 sideways, i.e. along the left/right axis and perpendicularly to the dorsoventral axis (Fig 1b) and only one implant per animal was inserted. Before insertion, implants were coated with a titaniferous layer of about 30 nm (pfm medical titanium gmbh, Nuremberg, Germany) and sterilized with autoclave. Implantation was performed under isoflurane anesthesia and the mice were sacrificed 6 weeks after implant insertion. All animal procedures were approved by the local authorities (Kantonales Veterinäramt Zürich, License No. 190/2010, Switzerland).

### *In vivo* micro-CT scans, image processing and measured parameters

The timeline of the *in vivo* imaging experiment is displayed in Fig 1a: the entire CV6 was scanned nine consecutive times using longitudinal *in vivo* micro-CT (vivaCT 40, Scanco Medical, Brüttisellen, Switzerland). The first two scans were performed before OVX/SHM surgery and 9 weeks after surgery (i.e., one day before implantation), respectively. The third scan was acquired right after implant insertion (week 0), and the remaining measurements were performed weekly for the following 6 weeks (week 1, week 2, etc.). The second and third scans were performed one day apart to measure structural parameters right before and after implant insertion, with the aim to monitor early structural changes due to implantation. The scanning procedure followed an optimized protocol for bone-implant systems [20]: images were acquired at a nominal isotropic voxel-size of 10.5 μm, with an integration time of 350 ms, 500 projections, 21 mm field of view and no frame averaging. The corresponding spatial resolution for an object positioned in the center of the field of view is 17.2 μm measured by the manufacturer as the 10% threshold of the modular transfer function (10% MTF). The peak voltage and the current of the micro-CT were set to 55 kVp and 145 μA, respectively. The total scanning time for imaging the entire vertebra with the implant was about 15 minutes. The estimated total dose delivered within the entire experiment was about 6 Gy [34] distributed over 15 weeks; based on previous knowledge of radiation effects, such a dose is not expected to cause large modifications of bone architecture and (re)modeling [35–37].

**Fig 2:**
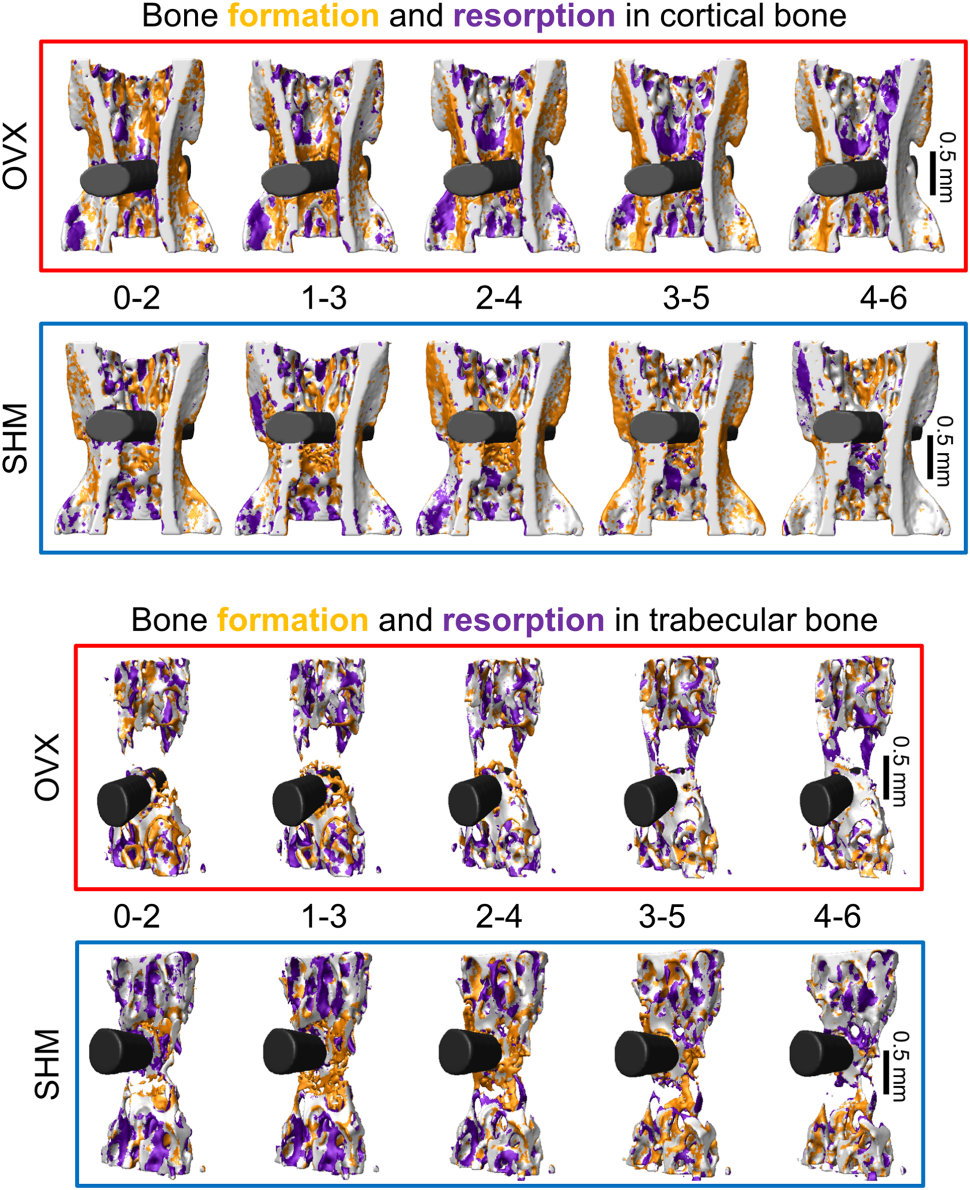
Three-dimensional visualization of the bone formation and bone resorption sites over time in cortical (top) and trabecular (bottom) bone for a representative ovariectomized (OVX) and sham-operated (SHM) mouse.

Image processing involved several steps (summarized in Fig 1c), which have been conceived and refined in earlier work on longitudinal *in vivo* micro-CT imaging of mouse vertebrae [37, 38]. The virtual reconstructions of the vertebra were first aligned along the cranio-caudal axis [39]. Three dimensional regions of bone formation and bone resorption due to (re)modeling were obtained by registering two consecutive scans of the same vertebra with the implant within a two-week time interval (Fig 2). Specifically, images were registered using a rigid intensity-based, least-squared registration method [40], with interpolation algorithm based on B-Splines [39]. We did not register on the basis of the implant alone as, especially in the very early time points following insertion, the implant could still slightly move due to the daily activity of the animal. Even if small, those movements would cause a systematic shift of the measured rates of bone formation and resorption towards too large values. By registering the entire implanted bone, the effect of possible implant movements on remodeling rates was minimized and the presence of the implant did not increase the typical registration error of the procedure [20]. After registration, a Gaussian filter (support 1, sigma 1.2) was applied to the grey-scale images to reduce the noise. A global fixed threshold, corresponding to 560 mg HA/cm^3^ (or 31.6% of the maximum grey value), was used to separate the bone-implant system from the background (Fig 1c, left). Bone voxels only present in the first scan were considered resorbed bone, while voxels only present in the second scan were consider formed bone. Single formed and resorbed voxels were discarded from the analysis to decrease registration and partial volume errors [20]. Cortical and trabecular bone were detected in a fully automatic manner with an algorithm described elsewhere [37, 41] while the implant was separated from the surrounding bone using a semi-automatic approach which involved minimal user interaction [20]. The growth plates were identified as previously reported [37] and excluded from the analysis. The amount of bone (expressed in percentage of the total length of the vertebra) which was not analyzed as being part of the growth plates was 20% and 25% for trabecular and cortical compartment, respectively.

Within the cortical compartment, we distinguished between periosteal and endocortical surfaces, which were identified by extracting the outer and inner surface voxels of the cortical shell, respectively (Fig 1c, right). Being interested in the changes close to and far from the implant, the different bone regions analyzed (i.e., trabecular, periosteal and endocortical bone) were further divided into a peri-implant (i.e., bone voxels located up to 420 μm away from the implant surface) and a distant (i.e., bone voxels situated more than 420 μm away from the implant surface) region (Fig 1c, left). The value of 420 μm (which is close to the value of implant diameter) was chosen to include in peri-implant bone the damaged zone induced by implant insertion, which was visually detectable in the micro-CT scans [20]. Depending on implant dimension, bone type and insertion procedure, microdamage regions ranging from 100 to 1000 μm have been reported [14, 16, 21].

Bone architecture was characterized by measuring full tissue volume (TV), full vertebral length (VL) along the cranio-caudal axis of the entire CV6 (including growth plates), cortical thickness (Ct.Th), cortical area fraction (Ct.Ar/Tt.Ar), cortical porosity (Ct.Po), trabecular bone volume fraction (BV/TV), trabecular thickness (Tb.Th) and trabecular number (Tb.N). Those parameters were measured right before OVX/SHM surgery, 9 weeks after surgery and for the 6 consecutive weeks after implantation. Bone (re)modeling was characterized by bone formation rate (BFR), bone resorption rate (BRR) and net rate of bone (re)modeling (i.e., BFR minus BRR). Formation and resorption rates were computed based on formed and resorbed voxels [37] within a two-week time interval (Fig 2), which allowed the occurrence of sufficient changes for accurate detection with the current image resolution of the *in vivo* micro-CT [20]. At the periosteal and endocortical surfaces, BFR and BRR were normalized to the actual bone surface (BS) while in the trabecular compartment they were normalized to the present bone volume (BV). Net (re)modeling rate was computed by subtracting BRR to BFR with the corresponding normalizations. All architectural and (re)modeling parameters were calculated and reported according to standard guidelines [42].

### Statistics

Architectural and (re)modeling parameters were tested for significant differences between OVX and SHM using the Mann-Whitney Rank Sum Test as the two groups did not always follow a normal distribution indicated by the Kolmogorov-Smirnov normality test nor did they always have equal variance in each time point. The Mann-Whitney Rank Sum Test is considered a suitable alternative to ANOVA when the assumption for normality and equal variance of the data cannot be met. Since during the monitoring time, the two groups did not receive any specific repeated treatment (e.g. mechanical loading or drug administration) we were not concerned about possible interactions among multiple factors. The same test was used to investigate differences between periosteal and endocortical surface within OVX and SHM mice. To test the significance between the first and last time point within the same group a paired t-test was used after verifying normal distribution of the data; a Wilcoxon Signed Rank Test was applied otherwise. P-values smaller than 0.05 were considered significant and all data are shown as mean ± standard error.

## Results

### Structural modification after OVX/SHM surgery and before implantation

Ovary removal deteriorated the structure of both cortical and trabecular bone in the mouse caudal vertebra CV6 (Table 1). Nine weeks after surgery, cortical thickness decreased by ~7% in OVX mice, in contrast with the behavior of SHM, where an increase of practically the same magnitude was observed. The cortex in OVX animals was not only thinner but also more porous (around 14%) whereas Ct.Po in SHM mice diminished of about 10%. The periosteal surface expanded considerably in OVX (13%) but stayed practically constant in SHM. The endocortical surface showed a minor increase in the OVX group (around 3.5%), whereas it decreased by 7% in SHM animals. The loss of trabecular bone volume fraction (BV/TV) due to OVX surgery was substantial and amounted to roughly 33%. The loss was caused by the trabeculae getting thinner and eventually even disappearing from the trabecular network. Conversely, SHM mice showed a pronounced increase in BV/TV of about 30% mainly due to trabecular thickening and not to an increase in trabecular number. One additional effect of OVX at the level of whole bone was an increase in vertebral length (VL, Table 1), with the relative increase 9 weeks after OVX surgery being significantly higher in OVX (almost 6%) when compared to SHM (~1.5%).

**Table 1:** Changes in bone architecture after estrogen removal.

Parameter	OVX			SHM		
	Before surgery	After surgery	Change [%]	Before surgery	After surgery	Change [%]
*Full bone*						
TV [mm^3^]	6.98 ± 0.19	7.63 ± 0.19	9.34 ± 0.66 #	7.19 ± 0.16	7.51 ± 0.17	4.49 ± 0.22 #
VL [mm]	3.87 ± 0.03	4.10 ± 0.03	5.91 ± 0.32 #	3.86 ± 0.04	3.92 ± 0.04 *	1.55 ± 0.18 #
*Cortical bone*						
Ct.Th [mm]	0.159 ± 0.002	0.147 ± 0.002	-7.39 ± 1.13 #	0.163 ± 0.002	0.173 ± 0.003 *	7.47 ± 0.97 #
Ct.Ar/Tt.Ar [%]	43.77 ± 0.47	38.74 ± 0.64	-11.43 ± 1.55 #	44.69 ± 0.57	47.52 ± 0.74 *	6.37 ± 1.18 #
Ct.Po [%]	17.47 ± 0.25	19.95 ± 0.46	14.20 ± 2.20 #	16.93 ± 0.21	15.16 ± 0.26 *	-10.43 ± 1.05 #
Periosteal surface [mm^2^]	16.82 ± 0.34	19.02 ± 0.47	13.01 ± 0.83 #	16.12 ± 0.35	16.18 ± 0.34 *	0.43 ± 0.34
Endocortical surface [mm^2^]	12.76 ± 0.35	13.20 ± 0.39	3.45 ± 0.74 #	12.15 ± 0.25	11.25 ± 0.15 *	-7.32 ± 0.94 #
*Trabecular bone*						
BV/TV [%]	15.67 ± 0.61	10.37 ± 0.40	-33.35 ± 2.58 #	16.93 ± 0.63	21.90 ± 0.51 *	30.10 ± 3.51 #
Tb.Th [mm]	0.076 ± 0.001	0.068 ± 0.001	-11.02 ± 2.17 #	0.080 ± 0.001	0.094 ± 0.002 *	17.68 ± 1.95 #
Tb.N [1/mm]	2.43 ± 0.07	2.17 ± 0.04	-10.42 ± 1.62 #	2.51 ± 0.07	2.56 ± 0.07 *	2.02 ± 1.23

Bone architectural parameters before surgery and 9 weeks after surgery (i.e., one day before implant insertion) for ovariectomized (OVX) and sham-ovariectomized (SHM) mice. Percentage changes were calculated for each animal and averaged.

^#^ denotes a significant difference (p < 0.05) before and after surgery within the same group.

* denotes a significant difference (p < 0.05) between groups within the same time point. Data reported as mean ± standard error.

### Time course of bone (re)modeling close to the implant

#### Cortical bone

**Fig 3:**
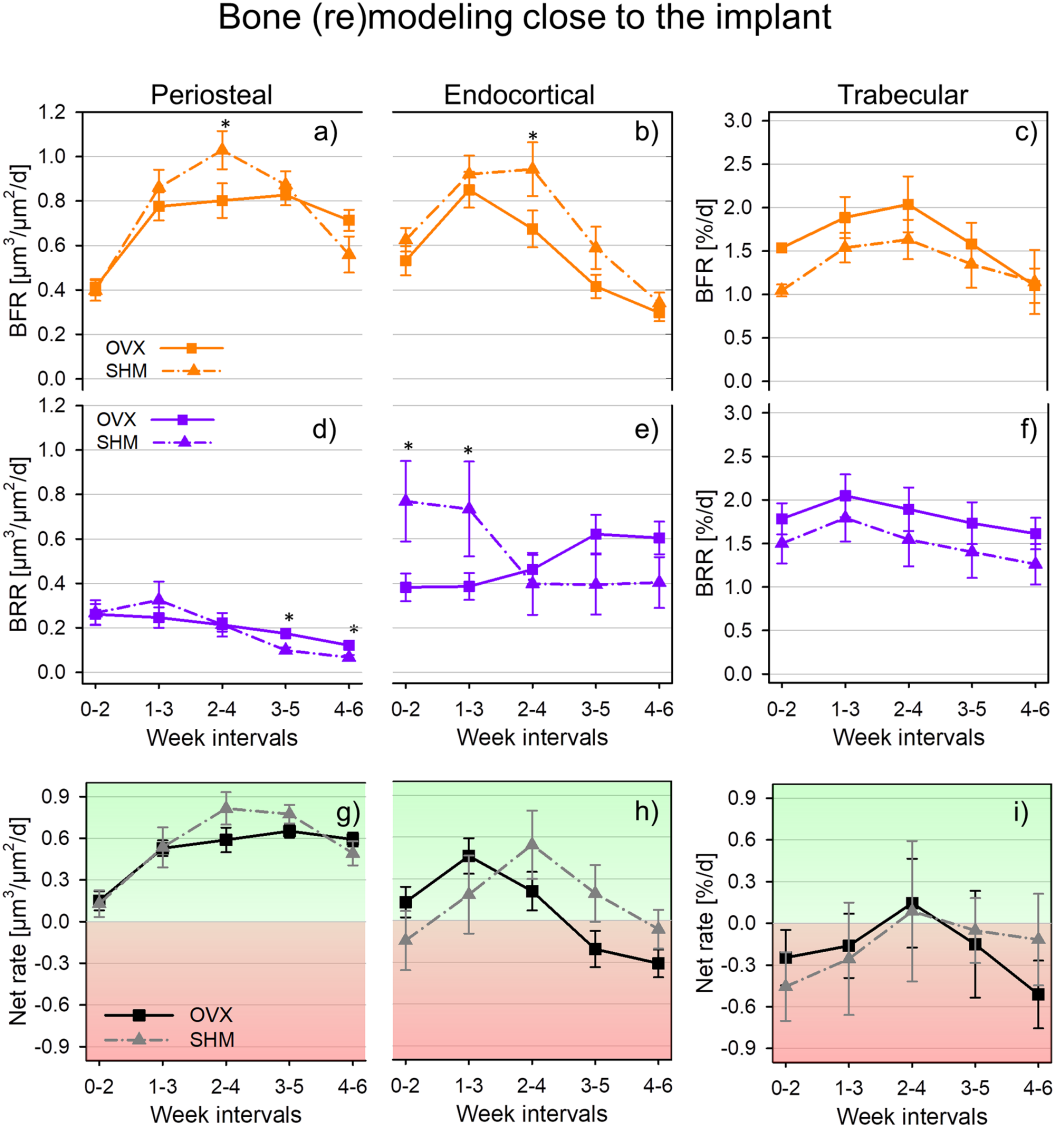
Bone (re)modeling rates for ovariectomized (OVX) and sham-ovariectomized (SHM) mice in peri-implant region of periosteal surface (a, d and g), endocortical surface (b, e and h), and trabecular bone (c, f and i). * denotes a significant difference (p < 0.05) between groups within the same time interval. Significant differences between first and last time interval are marked by # in Table 2. Data reported as mean ± standard error.

A common feature of both periosteal and endocortical surfaces of peri-implant cortical bone is that bone formation rate (BFR) increased transiently after implant insertion as indicated by the peak-shaped curves of both SHM and OVX animals (Fig 3a and 3b). In SHM, the maximum values of BFR occurred always at week interval 2–4 (i.e., from two to four weeks after implantation): there, the relative increase in BFR with respect to week interval 0–2 (i.e., right after implantation) was about 155% and 52% for periosteal and endocortical surface, respectively. In week interval 2–4, cortical BFR in SHM was significantly higher than in OVX (29% for periosteal surface and 40% for endocortical surface). In all the remaining week intervals, BFR values of SHM and OVX were not statistically different. The behavior of peri-implant bone resorption was fairly dissimilar between the two surfaces analyzed (Fig 3d and 3e): periosteal BRR (Fig 3d) was always significantly smaller than endocortical BRR (Fig 3e); at the end of the monitoring period (i.e., week interval 4–6), only a minimal amount of periosteal bone resorption was detected with, however, significantly higher values for OVX than SHM. Conversely, a high BRR was measured at endocortical bone but only in SHM animals and soon after implant placement: for instance, in week interval 0–2 BRR in SHM was roughly a factor of two higher than in OVX. In general, BRR in SHM had a decreasing trend whereas the opposite was observed for OVX, although time changes between initial and final BRR were not statistically significant (see also Table 2). Considering net (re)modeling rate at the periosteal surface (Fig 3g) both SHM and OVX mice had bone formation slightly prevailing over bone resorption, and implantation tipped the balance even further towards bone formation with the relative increase in net (re)modeling being almost a factor of four and quite similar between SHM and OVX (Table 2). Endocortical net bone (re)modeling was somewhat dephased in time when comparing SHM and OVX (Fig 3h): there was a positive peak occurring at week interval 1–3 in OVX and at week interval 2–4 in SHM. At the end of the monitoring period, both groups had a slight imbalance towards bone resorption.

**Table 2:** Bone (re)modeling post implantation close to the implant.

Parameter	OVX			SHM		
	Week 0	Week 6	Variation [%]	Week 0	Week 6	Variation [%]
*Periosteal*						
BFR [μm^3^/μm^2^/d]	0.41 ± 0.04	0.71 ± 0.05	83.13 ± 18.35 #	0.40 ± 0.05	0.56 ± 0.08	24.38 ± 16.86
BRR [μm^3^/μm^2^/d]	0.26 ± 0.05	0.12 ± 0.02	-46.29 ± 13.95 #	0.27 ± 0.06	0.06 ± 0.01	-68.26 ± 5.74 #
Net rate [μm^3^/μm^2^/d]	0.15 ± 0.07	0.59 ± 0.05	266.8 ± 98.57 #	0.13 ± 0.10	0.49 ± 0.09	210.3 ± 58.77 #
*Endocortical*						
BFR [μm^3^/μm^2^/d]	0.53 ± 0.07	0.30 ± 0.04	-40.65 ± 17.68 #	0.63 ± 0.05	0.34 ± 0.05	-38.27 ± 14.08 #
BRR [μm^3^/μm^2^/d]	0.38 ± 0.06	0.60 ± 0.07	50.83 ± 18.19	0.77 ± 0.18 *	0.40 ± 0.11	-35.34 ± 20.51
Net rate [μm^3^/μm^2^/d]	0.15 ± 0.10	-0.31 ± 0.10	-215.3 ± 79.57 #	-0.14 ± 0.21	-0.06 ± 0.14	52.3 ± 21.13
*Trabecular*						
BFR [%/d]	1.53 ± 0.05	1.10 ± 0.20	-26.14 ± 14.71	1.05 ± 0.07	1.14 ± 0.37	13.83 ± 37.51
BRR [%/d]	1.78 ± 0.18	1.61 ± 0.18	-3.27 ± 14.71	1.50 ± 0.23	1.26 ± 0.23	-10.67 ± 13.39
Net rate [%/d]	-0.25 ± 0.20	-0.51 ± 0.24	-91.65 ± 77.31	-0.45 ± 0.25	-0.12 ± 0.33	51.99 ± 14.43

Bone (re)modeling rates post implantation (week interval 0–2) and at the end of the monitoring period (week interval 4–6) for ovariectomized (OVX) and sham-ovariectomized (SHM) mice. Percentage changes were calculated for each animal and averaged.

^#^ denotes a significant difference (p < 0.05) between first and last week interval within the same group.

* denotes a significant difference (p < 0.05) between groups within the same time interval. Data reported as mean ± standard error.

#### Trabecular bone

In the peri-implant trabecular bone, (re)modeling rates of OVX and SHM were not statistically different (Fig 3c and 3f). Both groups showed a small peak in BRR at week interval 1–3 (Fig 3f) followed, one week later, by a corresponding (limited) increase in BFR (Fig 3c). In general, OVX animals had slightly higher (re)modeling rates throughout the entire monitoring period, with the exception of BFR in the last week interval. Net bone (re)modeling was predominantly negative in both groups, indicating prevailing bone resorption not only at the early stages post implantation but also 6 weeks later (Fig 3i).

### Time course of bone (re)modeling far from the implant

#### Cortical bone

**Fig 4:**
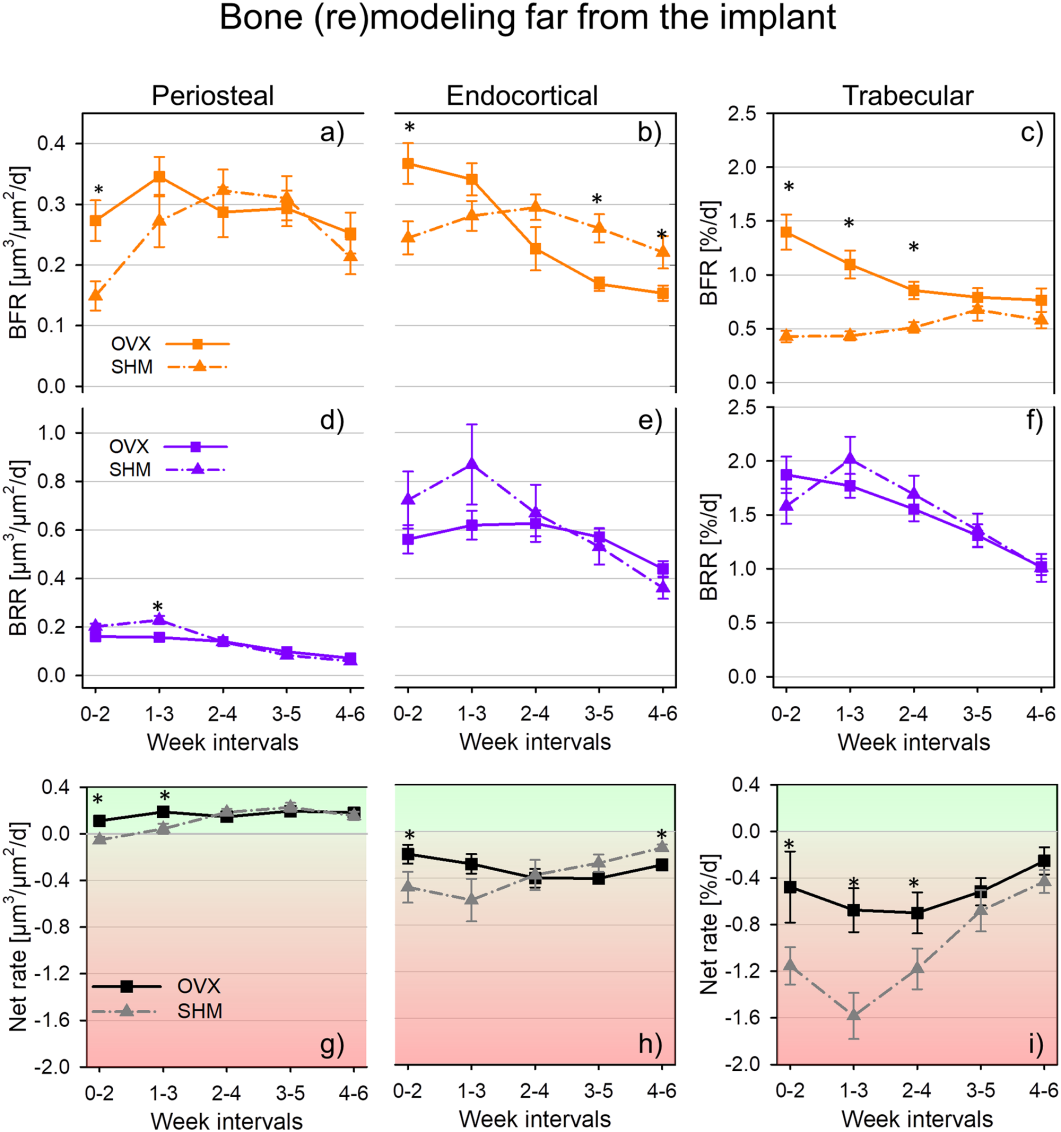
Bone (re)modeling rates for ovariectomized (OVX) and sham-ovariectomized (SHM) mice in the distant region of periosteal surface (a, d and g), endocortical surface (b, e and h), and trabecular bone (c, f and i). * denotes a significant difference (p < 0.05) between groups within the same time interval. Significant differences between first and last time interval are marked by # in Table 3. Data reported as mean ± standard error.

In the region far from the implant, (re)modeling rates of SHM and OVX had substantially different time courses, especially considering bone formation (Fig 4). In the first week interval post implantation, the absolute values of both periosteal and endocortical BFR in OVX mice were about 80% and 50% higher than in SHM (Fig 4a and 4b). In SHM mice, however, the trajectories of BFR peaked in week interval 2–4 on both inner and outer surfaces (as observed also close to the implant), with the relative increase with respect to the initial week interval being much higher for periosteal (113%) than for endocortical (21%) bone. BFR values in the last week interval were either similar to initial BFR (endocortical surface) or still higher (periosteal surface) (Table 3). A comparable increasing trend in BFR after implant insertion was not observed in OVX mice: endocortically BFR even substantially decreased (roughly 54% when comparing the first and last week interval) whereas at the periosteal surface the trend was less regular, showing a peak at week interval 1–3 and with initial and final values being not significantly different (Table 3). Regarding cortical bone resorption, the major distinctions between SHM and OVX were concentrated within the first two week intervals following implantation where SHM mice had a slightly higher BRR than OVX and always showed a well-defined peak in the second week interval corresponding to a relative increase in BRR with respect to the initial value of 15% for periosteal (Fig 4d) and 21% for endocortical (Fig 4e) bone. The difference between SHM and OVX got smaller towards the end of the monitoring period as indicated by the overlapping of the two time courses with no significant differences between the two groups after week interval 1–3 (Fig 4d and 4e). In general, BRR decreased significantly in both groups and in both surfaces with the larger decrease (about 70%) observed in SHM at the periosteal location (Table 3). Additionally, periosteal BRR (Fig 4d) was always at least a factor of two smaller than endocortical BRR (Fig 4e). Post implantation in the distant cortical bone, (re)modeling balance was predominantly positive at the periosteal location (Fig 4g) but always negative at the endocortical (Fig 4h) surface. There were significant differences in net (re)modeling rate between SHM and OVX mice, with OVX having significantly higher values for either one (endocortical bone, Fig 4h) or two (periosteal bone, Fig 4g) week intervals following implant insertion. At the end of the experiment, only the (re)modeling balance at the endocortical surface was still significantly different between SHM and OVX with the former having slightly smaller values indicating less net bone resorption (Fig 4h).

**Table 3:** Bone (re)modeling post implantation far from the implant.

Parameter	OVX			SHM		
	Week 0	Week 6	Variation [%]	Week 0	Week 6	Variation [%]
*Periosteal*						
BFR [μm^3^/μm^2^/d]	0.27 ± 0.03	0.25 ± 0.01	2.01 ± 14.50	0.15 ± 0.02 *	0.21 ± 0.03	34.92 ± 20.02
BRR [μm^3^/μm^2^/d]	0.16 ± 0.02	0.07 ± 0.01	-50.24 ± 10.44 #	0.20 ± 0.01	0.06 ± 0.01	-69.68 ± 6.98 #
Net rate [μm^3^/μm^2^/d]	0.11 ± 0.04	0.18 ± 0.04	65.02 ± 43.93	-0.05 ± 0.03 *	0.15 ± 0.03	275.0 ± 85.32 #
*Endocortical*						
BFR [μm^3^/μm^2^/d]	0.36 ± 0.03	0.15 ± 0.01	-54.15 ± 6.50 #	0.24 ± 0.03 *	0.22 ± 0.03	-13.15 ± 17.24
BRR [μm^3^/μm^2^/d]	0.56 ± 0.06	0.44 ± 0.03	-15.04 ± 10.45	0.72 ± 0.12	0.36 ± 0.04	-46.25 ± 6.74 #
Net rate [μm^3^/μm^2^/d]	-0.19 ± 0.08	-0.29 ± 0.04	-114.9 ± 83.74	-0.48 ± 0.13 *	-0.14 ± 0.03 *	64.74 ± 8.81 #
*Trabecular*						
BFR [%/d]	1.40 ± 0.16	0.76 ± 0.11	-35.80 ± 12.43 #	0.43 ± 0.05 *	0.58 ± 0.08	28.16 ± 19.03
BRR [%/d]	1.87 ± 0.17	1.02 ± 0.07	-42.83 ± 5.21 #	1.58 ± 0.16	1.01 ± 0.13	-29.35 ± 13.90 #
Net rate [%/d]	-0.48 ± 0.31	-0.25 ± 0.12	50.73 ± 31.84	-1.15 ± 0.16 *	-0.43 ± 0.10	65.95 ± 8.58 #

Bone (re)modeling rates post implantation (week interval 0–2) and at the end of the monitoring period (week interval 4–6) for ovariectomized (OVX) and sham-ovariectomized (SHM) mice. Percentage changes were calculated for each animal and averaged.

^#^ denotes a significant difference (p < 0.05) between first and last week interval within the same group.

* denotes a significant difference (p < 0.05) between groups within the same time interval. Data reported as mean ± standard error.

#### Trabecular bone

Post implantation, OVX and SHM had also substantially different behaviors in the rate of trabecular bone formation far from the implant. At early stages after implantation, BFR in OVX was up to a factor of three higher than in SHM (week interval 0–2, Fig 4c). Such initially elevated BFR decreased with time (about 39% already after three week intervals) whereas in SHM mice did not change significantly with time (Table 3). At the end of the monitoring period, similar BFR were observed for both groups. Bone resorption had a well-defined decreasing time course, with the exception of a peak observed in SHM at week interval 1–3 where BRR showed a relative increase of about 27% compared to the initial week interval (Fig 4f). Similar to the cortical region, the difference between groups became progressively smaller towards the end of the monitoring period; however, no significant differences between groups were detected in any of the week intervals considered (Fig 4f). (Re)modeling balance in the distant trabecular bone was always negative, with the net (re)modeling rate in SHM being significantly higher (in absolute values) than in OVX up to the mid time point of the experiment (Fig 4i) and indicating a scenario vastly dominated by bone loss. However, this difference was no longer present in the last two week intervals of the monitoring period.

### Architectural changes close to and far from the implant

**Fig 5:**
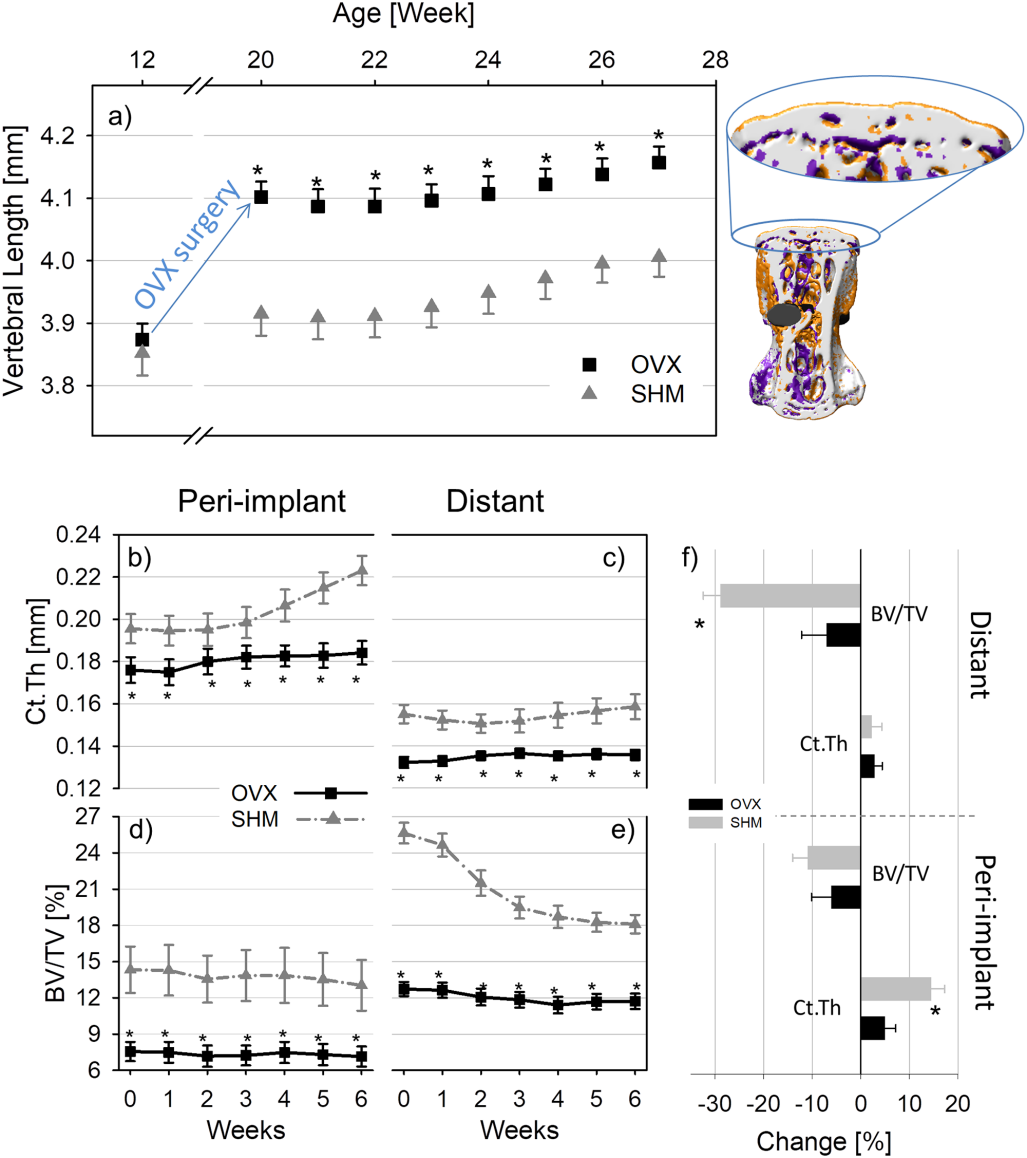
Changes in the length of the implanted vertebra monitored for the entire experiment (including OVX/SHM surgery and implant insertion). On the right, a three-dimensional visualization of bone remodeling in the whole vertebra is reported, with the lengthening corresponding to high bone formation localized in the growth plates (which were excluded from the analysis of bone (re)modeling) (a). Time evolution of cortical thickness (Ct.Th) and bone volume fraction (BV/TV) in the peri-implant (b, d) and distant bone (c, e) for ovariectomized (OVX) and sham-ovariectomized (SHM) mice following implantation. Differences in Ct.Th and BV/TV between the first and the last time point in the peri-implant and distant region are also shown in percentage changes (f). * denotes a significant difference (p < 0.05) between groups. Significant differences between first and last time point are marked by # in Table 4. Data reported as mean ± standard error.

Changes in the (re)modeling process following implant insertion caused substantial modification in bone architecture (Fig 5 and Table 4). Peri-implant Ct.Th in SHM started to increase (linearly) three weeks after implantation (Fig 5b) with a speed of around 8 μm/week, and at the end of the experiment (i.e. week 6) it was higher than at week 0 of about 14% (Fig 5f). Ct.Th in OVX right after implantation was about 15% smaller than in SHM and also showed a slight increase, although the difference between week 0 and week 6 was not statistically significant (Fig 5f). Close to the implant, the generally negative (re)modeling balance in trabecular bone was responsible for the progressive bone loss (Fig 5d) which amounted to 6% in OVX and 11% in SHM when comparing week 0 to week 6 (Fig 5f). Noteworthy, the initial amount of trabecular bone around the implant was almost a factor of two larger in SHM (~14%) than OVX (~7.5%).

**Table 4:** Changes in bone architecture following implant placement.

Parameter	OVX			SHM		
	Week 0	Week 6	Change [%]	Week 0	Week 6	Change [%]
*Full bone*						
TV [mm^3^]	7.48 ± 0.19	8.00 ± 0.16	7.10 ± 0.70 #	7.27 ± 0.16	7.76 ± 0.18	6.85 ± 0.45 #
VL [mm]	4.09 ± 0.03	4.16 ± 0.03	1.72 ± 0.31 #	3.91 ± 0.04 *	4.01 ± 0.03 *	2.49 ± 0.31 #
***Peri-implant region***						
*Cortical bone*						
Ct.Th [mm]	0.176 ± 0.006	0.184 ± 0.006	4.98 ± 2.20 #	0.196 ± 0.007 *	0.223 ± 0.007 *	14.43 ± 2.80 #
Ct.Ar/Tt.Ar [%]	43.35 ± 1.43	44.42 ± 1.45	2.90 ± 3.09	50.64 ± 1.40 *	54.12 ± 1.86 *	7.27 ± 4.05
Ct.Po [%]	23.77 ± 0.81	23.38 ± 0.77	-1.37 ± 2.57	21.61 ± 0.67	18.55 ± 0.64 *	-13.70 ± 3.81 #
*Trabecular bone*						
BV/TV [%]	7.55 ± 0.80	7.15 ± 0.83	-6.07 ± 4.08	14.33 ± 1.97 *	13.04 ± 2.10 *	-10.98 ± 3.03 #
Tb.Th [mm]	0.074 ± 0.002	0.083 ± 0.002	11.72 ± 3.38 #	0.089 ± 0.003 *	0.104 ± 0.004 *	17.37 ± 3.62 #
Tb.N [1/mm]	2.22 ± 0.10	1.98 ± 0.15	-10.90 ± 4.65	2.57 ± 0.13	2.20 ± 0.13	-14.74 ± 2.31 #
SMI [–]	2.01 ± 0.11	1.86 ± 0.15	-7.40 ± 6.23	1.71 ± 0.19	1.68 ± 0.21	0.32 ± 7.37
***Distant region***						
*Cortical bone*						
Ct.Th [mm]	0.132 ± 0.003	0.136 ± 0.002	2.83 ± 1.57	0.155 ± 0.004 *	0.159 ± 0.006 *	2.25 ± 2.09
Ct.Ar/Tt.Ar [%]	40.74 ± 0.64	39.14 ± 0.72	-3.83 ± 1.83	49.56 ± 1.06 *	46.66 ± 1.74 *	-5.84 ± 2.80
Ct.Po [%]	22.96 ± 0.49	23.04 ± 0.50	0.58 ± 2.38	17.69 ± 0.40 *	19.01 ± 0.74 *	7.28 ± 2.77 #
*Trabecular bone*						
BV/TV [%]	12.73 ± 0.59	11.71 ± 0.64	-7.08 ± 5.12	25.64 ± 0.86 *	18.11 ± 0.77 *	-28.93 ± 3.48 #
Tb.Th [mm]	0.068 ± 0.002	0.075 ± 0.001	9.41 ± 2.55 #	0.93 ± 0.002 *	0.090 ± 0.003 *	-3.05 ± 3.09
Tb.N [1/mm]	2.51 ± 0.02	2.19 ± 0.04	-12.70 ± 1.62 #	2.76 ± 0.09 *	2.55 ± 0.11 *	-6.57 ± 6.20

Bone architectural parameters at the first (week 0) and last (week 6) time point for ovariectomized (OVX) and sham-ovariectomized (SHM) mice. Percentage changes were calculated for each animal and averaged.

^#^ denotes a significant difference (p < 0.05) between first and last time point within the same group.

* denotes a significant difference (p < 0.05) between groups within the same time point. Data reported as mean ± standard error.

Considering bone located far from the implant surface, the different (re)modeling behaviors at the periosteal and endocortical surfaces resulted in a virtual steady-state cortical bone architecture, as indicated by a marginal increase in cortical thickness for both OVX and SHM, with no significant differences in the relative increase over time between groups (Fig 5f and Table 4). Again, at week 0 bone structure was weaker in OVX mice which had a cortex roughly 19% thinner than SHM (Fig 5c). Conversely, (re)modeling caused major structural changes in trabecular bone architecture far from the implant (Fig 5e), especially in SHM mice which suffered from a BV/TV loss of about 29% within a time period of 6 weeks (Fig 5f and Table 4). The loss was highest between week 1 and 3 (around 2%/week) and slowed down in the subsequent weeks (around 0.5%/week between week 4 and 6). The amount of BV/TV lost post-implantation in OVX was limited to 7% (Fig 5f and Table 4).

Considering whole bone changes, variations in vertebral length (VL) after implant placement were minor, with OVX and SHM showing similar trends (Fig 5a). In particular, relative changes in VL calculated in the same two-week time intervals used for assessing bone (re)modeling were always less than 0.85% in OVX and 1.2% in SHM. Interestingly, when comparing first (week 20) and last (week 27) time point, SHM animals showed a slightly larger increase in vertebral length than OVX (2.5% and 1.7%, respectively, Table 4).

## Discussion

In this study, we followed the changes in bone (re)modeling and bone architecture after implant insertion in healthy and estrogen-depleted mice. Although it is fairly clear that osteoporotic bone, with low bone mass and deteriorated bone architecture, provides a less favorable mechanical environment than healthy bone for implant fixation, there is no general agreement on the impact of osteoporosis on peri-implant bone (re)modeling, which is ultimately responsible for the long term stability of the bone-implant system. Here, using a mouse model mimicking estrogen deficiency-induced bone loss combined with longitudinal *in vivo* micro-CT, we investigated bone formation, bone resorption, and the consequent architectural modifications around the implant and in the whole implanted bone for 6 weeks following implantation. Specifically, we detailed the (re)modeling behavior on three different surfaces, which are known to respond differently to implant insertion [20, 28] as well as to osteoporosis [43, 44]. In general, trabecular bone is exposed to more (re)modeling events due to its bigger surface and thus suffers from a larger osteoporotic bone loss compared to cortical bone. Nevertheless, osteoporosis also alters cortical bone (re)modeling mainly by stimulating bone resorption at the inner endocortical surface without a corresponding increase in bone formation at the outer periosteal location, resulting in a net thinning of the cortical shell [43].

In our study, the main feature of peri-implant bone formation was a short—term acceleration of BFR in all the regions examined, with the relative increase in BFR being higher in healthy than estrogen depleted bone. Another study also reported a transient increase in formation rate post-implantation around the implant [28] but did not find clear-cut differences in (re)modeling rates between sham-operated and healthy rodents. One advantage of our approach is the possibility to measure (re)modeling in a direct three-dimensional way and to follow the very same animal over time (Fig 2). This may help to detect subtle differences which do not emerge from cross-sectional studies [21, 31, 37]. Considering peri-implant bone resorption, which we could assess directly from the longitudinal *in vivo* micro-CT images, a generally higher BRR was measured at the endocortical surface compared to periosteal bone in both groups. In genetically similar adult mice, periosteal resorption has been shown to be practically absent [44], therefore we could assume that implantation to some extent re-activate bone resorption at the periosteal surface, which, however, after one or two weeks decreased again to very low values, with SHM-operated mice having a faster and more pronounced decrease than OVX. Furthermore, BRR in SHM and OVX had rather different time course post-implantation also at the endocortical surface: a decreasing trend (starting from fairly high values) characterized SHM and a slight increasing behavior was measured for OVX. As previously noted [20], implantation had a long range effect, which involved the whole implanted vertebra, especially considering bone resorption. In fact, values of BFR in distant cortical bone were about 60% smaller than close to the implant whereas BRR had comparable magnitudes. This is in agreement with the results of Kettenberger and colleagues showing that modification in bone formation decayed faster than in bone resorption when moving away from the implant [21]. Far from the implant we could still detect a significant effect of estrogen removal, which again limited the relative increase in BFR post-implantation at the periosteal surface and, endocortically, caused a significantly lower BFR in OVX than SHM in later weeks. The fact that, at the initial time point, BFR but not BRR was significantly higher in OVX, may suggest that the transient increase in remodeling rates caused by estrogen removal (also referred to as remodeling transient [45]) was already over for bone resorption but not for formation. Overall, the reduced ability of OVX mice to increase peri-implant bone formation together with the delayed response in terms of peri-implant bone resorption is in line with previous results on fracture healing, which seems to be partially hindered in estrogen depleted animals [46].

One factor that could potentially contribute to (re)modeling differences between SHM and OVX may be the susceptibility to inflammatory reaction following implant insertion. Inflammation is a key player in the sequence of biological events occurring after implantation, especially in the early stages of the bone response [14]. Although the complex role of estrogen removal on the inflammation process is still not well grasped [47], there are indications that estrogen deficiency dysregulates bone (re)modeling by interacting with the immune system [48]. In our study, we only checked for obvious signs of inflammatory reaction such as swelling in the region around the implant, and did not find differences between SHM and OVX. However, as we did not measure markers of inflammation (which would have required a cross-sectional approach) we cannot exclude that the complex interplay between estrogen loss and inflammation state would have an impact on the (re)modeling behavior observed here. It is also known that OVX animals have the tendency to gain more weight than SHM: in a previous study on 15-week-old genetically identical mice, we found—5 weeks after surgery—a weight gain of about 23% and 11.5% for OVX and SHM animals, respectively [49]; whether weight differences per se could cause variations in the remodeling behavior is still an open question. Assuming that OVX and SHM animals would have the same level of physical activity, a higher weight would induce higher mechanical loading which, at least in healthy mice, would reduce bone resorption and increase bone formation [31, 37]. However, caudal vertebrae are not highly loaded skeletal locations and such effects, being superimposed to the well documented modifications in bone remodeling rates following OVX [32], cannot be clearly investigated with the present animal experiment. The (re)modeling behavior measured here is influenced by the metabolic state of the animal and its possible impact on bone growth. It is known that ovaries removal can increase not only animal weight but also vertebral length [32]. Our data showed that the largest increase in vertebral length occurred during a nine-week period following OVX/SHM surgery (Fig 5a). Conversely, after implant insertion, the variations in vertebral length in SHM and OVX were marginal and with similar trends. The increase in vertebral length was mainly due to high bone formation at the growth plates [32]: considering a nominal vertebral length of 4 mm, 1% increase in length would imply that each growth plate “moved” 20 μm, corresponding to roughly two voxels per week. Such small shift did not prevent our rigid registration approach, whereas much higher growth rates (e.g. after PTH administration) may require different strategies to superimpose the scans [50, 51]. Here, as the growth plates were excluded from the analysis of bone (re)modeling, the rather elevated bone formation at the growth plates did not bias the main results. In fact, after implant insertion, SHM animals showed a slightly higher increase in vertebral length than OVX. If such change in shape entered in our results, we would expect to see higher bone formation far from the implant (i.e. closer to the growth plates) for SHM. However, we measured exactly the opposite behavior, confirming that the effect of bone growth was ruled out by removing the growth plates.

Differences in bone (re)modeling were directly responsible for dissimilar modifications in peri-implant architecture; among all the architectural parameters measured (Table 4) we focused on the time course of Ct.Th and BV/TV as those two indices are most relevant for the mechanical competence of the vertebra [32, 52] and for the stability of the bone-implant system [8, 11]. The relative increase in cortical thickness was significantly higher in SHM than OVX mice: an initially higher BFR together with lower values of BRR towards the end of the study both contributed to the larger relative increase of cortical thickness in healthy mice. Such increase was mainly due to the expansion of the periosteal surface as indicated by the net periosteal and endocortical (re)modeling behavior. The adverse influence of hormonal status on peri-implant bone rearrangement confirms previous findings, which reported a decreased amount of peri-implant bone in estrogen depleted animals either in the initial days post implantation [29] or up to two weeks after implantation [24]. Here, we demonstrated that architectural differences between SHM and OVX were still present even six weeks after implant placement. The positive modifications in peri-implant cortical bone were contrasted by substantial bone loss, especially far from the implant and in SHM mice. There, the loss was caused by very low BFR, which could not counteract the high values of BRR. In SHM animals, the quick and substantial thickening of peri-implant cortical bone could have required bone to be resorbed elsewhere, for instance due to changes in the loading pattern. The interpretation might be that bone “shifts” from the trabecular network away from the implant to the cortical shell close to the implant to favor implant anchorage. Such “shift” may be hindered in OVX mice: in fact, considering the absolute values of BV/TV (Fig 5) it is clear that OVX animals had an initial bone volume fraction which was almost a factor of two smaller than SHM. Therefore, it may be that the bone volume is so low that, for the same level of physiological mechanical stimulations, the strains are much higher in OVX than SHM therefore preventing additional significant bone loss, which would further compromise whole vertebral bone strength.

There were limitations in the presented study that need to be mentioned. The statistical tests performed when data was not normally distributed may be less stringent than traditional ANOVA and, as a consequence, small differences that were identified as statistically significant should be interpreted with some caution. However, the main conclusions of our work are based on parameters which show high variations (i.e. always larger than 20%) between SHM and OVX at least in one time interval, namely: Ct.Th and BV/TV (Fig 5) as well as BFR and endocortical BRR (Figs 3 and 4). The resolution of the micro-CT is a limiting factor for detecting modification in trabecular thickness [53] as well as in the amount of formed/resorbed bone [30, 31]. Here, the minimum number of voxels that have to appear when superimposing two consecutive scans to be counted as formed/resorbed bone is one, with the additional constrains that the voxel must have in its closest neighborhood at least one additional voxel of the same type, i.e. identified as formed/resorbed. Image resolution also constrains the minimum time interval between two scans: assuming a mineral apposition rate of 2 μm per day [20], a minimum lag period of 5 days is required between two scans. We superimposed scans taken 14 days apart, therefore the average thickness of formed and resorbed bone packets is around 28 μm, which is above the nominal as well as true resolution of the micro-CT. The experimental error of our technique, mainly due to image registration (i.e., rotation of the coordinate system and interpolation) [39], has been previously quantified using repeated *ex vivo* measurements (including repositioning between the scans) of vertebrae measured after animal sacrifice and registered on the *in vivo* dataset: the quantity of erroneously formed/resorbed voxels per bone volume was less than 5% [1] and, in general, remodeling parameters could be assessed with fairly low precision errors similarly to standard architectural parameters [20, 30, 31, 54, 55]. It is worth mentioning that measurements of bone formation based on *in vivo* micro-CT of entire mice vertebrae as well as of specific regions of mice tibiae showed very high correlations with dynamic histomorphometry, which is considered gold standard to quantify bone remodeling [30, 31]. The mouse vertebral model used here may not adequately represent human cortical bone: adult mice are still growing in length while human cortical bone practically stops growing in early adulthood. Moreover, murine bone lacks osteons, which are pivotal features for the classical remodeling behavior of human cortical bone [56]: therefore, a cortical shell without osteons may not be fully representative for the behavior of thicker shells with osteons. Here, bone (re)modeling was monitored only after implantation, while the (re)modeling rates before implantation or without the implant were not characterized in the same animals. However, in a previous study, we compared the (re)modeling behavior of genetically identical mice of similar age with and without the implant and we found that in distant bone, formation was only marginally affected by the presence of the implant, whereas the same was not true for resorption [20]. Being mainly interested in the relative differences between OVX and SHM in bone (re)modeling post-implantation, the lack of a suitable control for bone (re)modeling should not affect the main conclusions of our study. In the present study we could not state whether bone changes induced by implant insertion and measured far from the implantation site would also appear in neighboring vertebrae (e.g., CV5 and CV7). Although there is evidence that during fracture repair and healing of large bone defects, a local acceleration of osteogenesis could trigger a systemic acceleration of bone formation [57, 58], in previous pilot studies we could not detect architecture and remodeling differences in CV6 when comparing scenarios with or without pins in neighbor vertebrae [33, 52, 59]. Likewise, the absence of effects on neighboring bones following the insertion of small pins was reported for a rat tail loading model [60, 61]. We also did not perform a direct mechanical assessment to characterize the impact of the observed architectural changes on implant anchorage. Destructive implant pull-out tests could be performed after animal sacrifice; however, considering that SHM bones had higher cortical thickness and bone volume fraction both close to and far from the implant, we would not expect mechanical testing to give much more insights than we could infer from the architectural changes. We did not directly asses bone implant contact, which is an important biomechanical parameter for (primary) implant stability. Although our approach in principle would allow measuring it, as metal artifacts were eliminated, we preferred to exclude surface voxels in contact with the implant from our analysis as those are most influenced by imaging and registration artifacts and therefore may have a grey value no representing the reality [20, 54] and typically overestimating the amount of bone in contact with the implant.

In conclusion, our hypotheses that OVX animals would have a decreased ability to modify bone (re)modeling and that the corresponding structural changes in peri—implant bone would be less effective for implant stability were confirmed in cortical bone. There, due to disturbed bone formation, estrogen depleted animals had a reduced capability to thicken the cortical shell in response to implant placement, which in turn indicates decreased implant fixation strength [11]. We also showed that the rapid increase in peri-implant cortical thickness observed in healthy animals may raise bone resorption elsewhere and, specifically, in the trabecular network far from the implant. The obtained knowledge on the dynamic response of diseased bone following implant insertion should provide useful guidelines to develop advanced treatments for osteoporotic fracture fixation which could be based, for instance, on selective manipulation of bone turnover in the peri-implant region by a local delivery of medications or by judicious administration of mechanical stimulation.

## Supporting information

S1 File NC3Rs ARRIVE guidelines checklist. (PDF)

## References

[1] Schulte FA, Ruffoni D, Lambers FM, Christen D, Webster DJ, Kuhn G, et al Local mechanical stimuli regulate bone formation and resorption in mice at the tissue level. Plos One. 2013;8(4):e62172 doi: 10.1371/journal.pone.0062172 ; 23637993 10.1371/journal.pone.0062172 PMC3634859

[2] Garnero P, SornayRendu E, Chapuy MC, Delmas PD. Increased bone turnover in late postmenopausal women is a major determinant of osteoporosis. J Bone Miner Res. 1996;11(3):337–49. doi: 10.1002/jbmr.5650110307 8852944 10.1002/jbmr.5650110307

[3] Fratzl P, Gupta HS, Paschalis EP, Roschger P. Structure and mechanical quality of the collagen-mineral nano-composite in bone. J Mater Chem. 2004;14(14):2115–23. doi: 10.1039/B402005g

[4] Ruffoni D, Fratzl P, Roschger P, Phipps R, Klaushofer K, Weinkamer R. Effect of temporal changes in bone turnover on the bone mineralization density distribution: A computer simulation study. J Bone Miner Res. 2008;23(12):1905–14. doi: 10.1359/jbmr.080711 18665790 10.1359/jbmr.080711

[5] Hernandez CJ, Keaveny TM. A biomechanical perspective on bone quality. Bone. 2006;39(6):1173–81. doi: 10.1016/j.bone.2006.06.001 16876493 10.1016/j.bone.2006.06.001 PMC1876764

[6] Farr JN, Khosla S. Skeletal changes through the lifespan-from growth to senescence. Nature Reviews Endocrinology. 2015;11(9):513–21. doi: 10.1038/nrendo.2015.89 26032105 10.1038/nrendo.2015.89 PMC4822419

[7] Goldhahn J, Suhm N, Goldhahn S, Blauth M, Hanson B. Influence of osteoporosis on fracture fixation—a systematic literature review. Osteoporosis Int. 2008;19(6):761–72. doi: 10.1007/s00198-007-0515-9 18066697 10.1007/s00198-007-0515-9

[8] Ruffoni D, Müller R, van Lenthe GH. Mechanisms of reduced implant stability in osteoporotic bone. Biomech Model Mechan. 2012;11(3–4):313–23. doi: 10.1007/s10237-011-0312-4 21562831 10.1007/s10237-011-0312-4

[9] Gabet Y, Kohavi D, Voide R, Mueller TL, Mueller R, Bab I. Endosseous implant anchorage is critically dependent on mechanostructural determinants of pen-implant bone trabeculae. J Bone Miner Res. 2010;25(3):575–83. doi: 10.1359/jbmr.090808 19653813 10.1359/jbmr.090808

[10] Basler SE, Traxler J, Muller R, van Lenthe GH. Peri-implant bone microstructure determines dynamic implant cut-out. Med Eng Phys. 2013;35(10):1442–9. doi: 10.1016/j.medengphy.2013.03.016 23623173 10.1016/j.medengphy.2013.03.016

[11] Ruffoni D, Wirth AJ, Steiner JA, Parkinson IH, Müller R, van Lenthe GH. The different contributions of cortical and trabecular bone to implant anchorage in a human vertebra. Bone. 2012;50(3):733–8. doi: 10.1016/j.bone.2011.11.027 22178777 10.1016/j.bone.2011.11.027

[12] Rocca M, Fini M, Giaveresi G, Aldini NN, Giardino R. Osteointegration of hydroxyapatite-coated and uncoated titanium screws in long-term ovariectomized sheep. Biomaterials. 2002;23(4):1017–23. doi: 10.1016/s0142-9612(01)00213-7 11791904 10.1016/s0142-9612(01)00213-7

[13] Marquezan M, Osorio A, Sant'Anna E, Souza MM, Maia L. Does bone mineral density influence the primary stability of dental implants? A systematic review. Clinical Oral Implants Research. 2012;23(7):767–74. doi: 10.1111/j.1600-0501.2011.02228.x 21635560 10.1111/j.1600-0501.2011.02228.x

[14] Marco F, Milena F, Gianluca G, Vittoria O. Peri-implant osteogenesis in health and osteoporosis. Micron. 2005;36(7–8):630–44. doi: 10.1016/j.micron.2005.07.008 16182543 10.1016/j.micron.2005.07.008

[15] Jenny G, Jauernik J, Bierbaum S, Bigler M, Gratz KW, Rucker M, et al A systematic review and meta-analysis on the influence of biological implant surface coatings on periimplant bone formation. J Biomed Mater Res A. 2016;104(11):2898–910. doi: 10.1002/jbm.a.35805 27301790 10.1002/jbm.a.35805

[16] Wang L, Ye T, Deng L, Shao J, Qi J, Zhou Q, et al Repair of microdamage in osteonal cortical bone adjacent to bone screw. Plos One. 2014;9(2):e89343 doi: 10.1371/journal.pone.0089343 24586702 10.1371/journal.pone.0089343 PMC3930719

[17] Recker R, Lappe J, Davies KM, Heaney R. Bone remodeling increases substantially in the years after menopause and remains increased in older osteoporosis patients. J Bone Miner Res. 2004;19(10):1628–33. doi: 10.1359/JBMR.040710 15355557 10.1359/JBMR.040710

[18] Tarantino U, Cerocchi I, Scialdoni A, Saturnino L, Feola M, Celi M, et al Bone healing and osteoporosis. Aging Clin Exp Res. 2011;23:62–4. 21970927

[19] Cheung WH, Miclau T, Chow SKH, Yang FF, Alt V. Fracture healing in osteoporotic bone. Injury. 2016;47:S21–S6. 10.1016/S0020-1383(16)47004-X 27338222

[20] Li Z, Kuhn G, von Salis-Soglio M, Cooke SJ, Schirmer M, Müller R, et al In vivo monitoring of bone architecture and remodeling after implant insertion: The different responses of cortical and trabecular bone. Bone. 2015;81:468–77. http://dx.doi.org/10.1016/j.bone.2015.08.017. 26303288 10.1016/j.bone.2015.08.017

[21] Kettenberger U, Ston J, Thein E, Procter P, Pioletti DP. Does locally delivered Zoledronate influence pen-implant bone formation?—Spatio-temporal monitoring of bone remodeling in vivo. Biomaterials. 2014;35(37):9995–10006. doi: 10.1016/j.biomaterials.2014.09.005 25241159 10.1016/j.biomaterials.2014.09.005

[22] Kurth AHA, Eberhardt C, Muller S, Steinacker M, Schwarz M, Bauss F. The bisphosphonate ibandronate improves implant integration in osteopenic ovariectomized rats. Bone. 2005;37(2):204–10. doi: 10.1016/j.bone.2004.12.017 15936997 10.1016/j.bone.2004.12.017

[23] Yamazaki M, Shirota T, Tokugawa Y, Motohashi M, Ohno K, Michi K, et al Bone reactions to titanium screw implants in ovariectomized animals. Oral Surg Oral Med O. 1999;87(4):411–8. doi: 10.1016/S1079-2104(99)70239-8 10.1016/s1079-2104(99)70239-8 10225622

[24] Chatterjee M, Hatori K, Duyck J, Sasaki K, Naert I, Vandamme K. High-frequency loading positively impacts titanium implant osseointegration in impaired bone. Osteoporosis Int. 2015;26(1):281–90. doi: 10.1007/s00198-014-2824-0 25164696 10.1007/s00198-014-2824-0

[25] Fini M, Aldini NN, Gandolfi MG, Belmonte MM, Giavaresi G, Zucchini C, et al Biomaterials for orthopedic surgery in osteoporotic bone: A comparative study in osteopenic rats. Int J Artif Organs. 1997;20(5):291–7. 9209931

[26] Fini M, Giavaresi G, Rimondini L, Giardino R. Titanium alloy osseointegration in cancellous and cortical bone of ovariectomized animals: Histomorphometric and bone hardness measurements. Int J Oral Max Impl. 2002;17(1):28–37. 11858572

[27] Vidigal GM, Groisman M, Gregorio LH, Soares GD. Osseointegration of titanium alloy and HA-coated implants in healthy and ovariectomized animals: a histomorphometric study. Clinical Oral Implants Research. 2009;20(11):1272–7. doi: 10.1111/j.1600-0501.2009.01739.x 19832768 10.1111/j.1600-0501.2009.01739.x

[28] Irish J, Virdi AS, Sena K, McNulty MA, Sumner DR. Implant placement increases bone remodeling transiently in a rat model. J Orthop Res. 2013;31(5):800–6. doi: 10.1002/jor.22294 23280449 10.1002/jor.22294

[29] Ozawa S, Ogawa T, Iida K, Sukotjo C, Hasegawa H, Nishimura RD, et al Ovariectomy hinders the early stage of bone-implant integration: Histomorphometric, biomechanical, and molecular analyses. Bone. 2002;30(1):137–43. 11792576 10.1016/s8756-3282(01)00646-9

[30] Schulte FA, Lambers FM, Kuhn G, Müller R. In vivo micro-computed tomography allows direct three-dimensional quantification of both bone formation and bone resorption parameters using time-lapsed imaging. Bone. 2011;48(3):433–42. doi: 10.1016/j.bone.2010.10.007 20950723 10.1016/j.bone.2010.10.007

[31] Birkhold AI, Razi H, Duda GN, Weinkamer R, Checa S, Willie BM. Mineralizing surface is the main target of mechanical stimulation independent of age: 3D dynamic in vivo morphometry. Bone. 2014;66:15–25. doi: 10.1016/j.bone.2014.05.013 24882735 10.1016/j.bone.2014.05.013

[32] Lambers FM, Kuhn G, Schulte FA, Koch K, Müller R. Longitudinal Assessment of In Vivo Bone Dynamics in a Mouse Tail Model of Postmenopausal Osteoporosis. Calcified Tissue Int. 2012;90(2):108–19. doi: 10.1007/s00223-011-9553-6 22159822 10.1007/s00223-011-9553-6

[33] Webster DJ, Morley PL, van Lenthe GH, Müller R. A novel in vivo mouse model for mechanically stimulated bone adaptation—a combined experimental and computational validation study. Comput Method Biomec. 2008;11(5):435–41. doi: 10.1080/10255840802078014 18612871 10.1080/10255840802078014

[34] Lambers FM, Kuhn G, Weigt C, Koch KM, Schulte FA, Müller R. Bone adaptation to cyclic loading in murine caudal vertebrae is maintained with age and directly correlated to the local micromechanical environment. J Biomech. 2015;48(6):1179–87. doi: 10.1016/j.jbiomech.2014.11.020 25543278 10.1016/j.jbiomech.2014.11.020

[35] Brouwers JEM, Van Rietbergen B, Huiskes R. No effects of in vivo micro-CT radiation on structural parameters and bone marrow cells in proximal tibia of Wistar rats detected after eight weekly scans. J Orthop Res. 2007;25(10):1325–32. doi: 10.1002/jor.20439 17568420 10.1002/jor.20439

[36] Willie BM, Birkhold AI, Razi H, Thiele T, Aido M, Kruck B, et al Diminished response to in vivo mechanical loading in trabecular and not cortical bone in adulthood of female C57Bl/6 mice coincides with a reduction in deformation to load. Bone. 2013;55(2):335–46. http://dx.doi.org/10.1016/j.bone.2013.04.023. 23643681 10.1016/j.bone.2013.04.023

[37] Lambers FM, Schulte FA, Kuhn G, Webster DJ, Müller R. Mouse tail vertebrae adapt to cyclic mechanical loading by increasing bone formation rate and decreasing bone resorption rate as shown by time-lapsed in vivo imaging of dynamic bone morphometry. Bone. 2011;49(6):1340–50. doi: 10.1016/j.bone.2011.08.035 21964411 10.1016/j.bone.2011.08.035

[38] Lambers FM, Koch K, Kuhn G, Ruffoni D, Weigt C, Schulte FA, et al Trabecular bone adapts to long-term cyclic loading by increasing stiffness and normalization of dynamic morphometric rates. Bone. 2013;55(2):325–34. doi: 10.1016/j.bone.2013.04.016 23624292 10.1016/j.bone.2013.04.016

[39] Schulte FA, Lambers FM, Mueller TL, Stauber M, Mueller R. Image interpolation allows accurate quantitative bone morphometry in registered micro-computed tomography scans. Comput Method Biomec. 2014;17(5):539–48. doi: 10.1080/10255842.2012.699526 22746535 10.1080/10255842.2012.699526

[40] Thevenaz P, Ruttimann UE, Unser M. A pyramid approach to subpixel registration based on intensity. Ieee Transactions on Image Processing. 1998;7(1):27–41. doi: 10.1109/83.650848 18267377 10.1109/83.650848

[41] Kohler T, Stauber M, Donahue LR, Müller R. Automated compartmental analysis for high-throughput skeletal phenotyping in femora of genetic mouse models. Bone. 2007;41(4):659–67. doi: 10.1016/j.bone.2007.05.018 17662679 10.1016/j.bone.2007.05.018

[42] Bouxsein ML, Boyd SK, Christiansen BA, Guldberg RE, Jepsen KJ, Müller R. Guidelines for assessment of bone microstructure in rodents using micro-computed tomography. J Bone Miner Res. 2010;25(7):1468–86. doi: 10.1002/jbmr.141 20533309 10.1002/jbmr.141

[43] Szulc P, Seeman E, Duboeuf F, Sornay-Rendu E, Delmas PD. Bone fragility: Failure of periosteal apposition to compensate for increased endocortical resorption in postmenopausal women. J Bone Miner Res. 2006;21(12):1856–63. doi: 10.1359/jbmr.060904 17002580 10.1359/jbmr.060904

[44] Birkhold AI, Razi H, Duda GN, Weinkamer R, Checa S, Willie BM. The Periosteal Bone Surface is Less Mechano-Responsive than the Endocortical. Sci Rep-Uk. 2016;6 ARTN 23480 doi: 10.1038/srep23480 27004741 10.1038/srep23480 PMC4804282

[45] Heaney RP. Remodeling and skeletal fragility. Osteoporosis Int. 2003;14:S12–S5. doi: 10.1007/s00198-003-1466-4 14504700 10.1007/s00198-003-1466-4

[46] Namkung-Matthai H, Appleyard R, Jansen J, Lin JH, Maastricht S, Swain M, et al Osteoporosis influences the early period of fracture healing in a rat osteoporotic model. Bone. 2001;28(1):80–6. doi: 10.1016/S8756-3282(00)00414-2 11165946 10.1016/s8756-3282(00)00414-2

[47] Weitzmann MN, Pacifici R. Estrogen deficiency and bone loss: an inflammatory tale. J Clin Invest. 2006;116(5):1186–94. doi: 10.1172/JCI28550 16670759 10.1172/JCI28550 PMC1451218

[48] Cenci S, Weitzmann MN, Roggia C, Namba N, Novack D, Woodring J, et al Estrogen deficiency induces bone loss by enhancing T-cell production of TNF-alpha. J Clin Invest. 2000;106(10):1229–37. doi: 10.1172/JCI11066 11086024 10.1172/JCI11066 PMC381439

[49] Weigt C. Interaction of mechanical loading with osteoporosis treatment in ovariectomized mice: Zürich; 2013.

[50] Altman AR, Tseng WJ, de Bakker CMJ, Chandra A, Lan SH, Huh BK, et al Quantification of skeletal growth, modeling, and remodeling by in vivo micro computed tomography. Bone. 2015;81:370–9. doi: 10.1016/j.bone.2015.07.037 26254742 10.1016/j.bone.2015.07.037 PMC4641023

[51] Lu YT, Boudiffa M, Dall'Ara E, Bellantuono I, Viceconti M. Development of a protocol to quantify local bone adaptation over space and time: Quantification of reproducibility. J Biomech. 2016;49(10):2095–9. doi: 10.1016/j.jbiomech.2016.05.022 27262181 10.1016/j.jbiomech.2016.05.022

[52] Webster D, Wasserman E, Ehrbar M, Weber F, Bab I, Müller R. Mechanical loading of mouse caudal vertebrae increases trabecular and cortical bone mass-dependence on dose and genotype. Biomech Model Mechan. 2010;9(6):737–47. doi: 10.1007/s10237-010-0210-1 20352279 10.1007/s10237-010-0210-1

[53] Verdelis K, Lukashova L, Atti E, Mayer-Kuckuk P, Peterson MG, Tetradis S, et al MicroCT morphometry analysis of mouse cancellous bone: intra- and inter-system reproducibility. Bone. 2011;49(3):580–7. doi: 10.1016/j.bone.2011.05.013 ; 21621659 10.1016/j.bone.2011.05.013 PMC3391301

[54] Lukas C, Ruffoni D, Lambers FM, Schulte FA, Kuhn G, Kollmannsberger P, et al Mineralization kinetics in murine trabecular bone quantified by time-lapsed in vivo micro-computed tomography. Bone. 2013;56(1):55–60. doi: 10.1016/j.bone.2013.05.005 23684803 10.1016/j.bone.2013.05.005

[55] Jariwala SH, Wee H, Roush EP, Whitcomb TL, Murter C, Kozlansky G, et al Time course of peri-implant bone regeneration around loaded and unloaded implants in a rat model. J Orthop Res. 2016:n/a–n/a. doi: 10.1002/jor.23360 . 27381807 10.1002/jor.23360 PMC5800527

[56] Parfitt AM. Osteonal and Hemi-Osteonal Remodeling—the Spatial and Temporal Framework for Signal Traffic in Adult Human Bone. J Cell Biochem. 1994;55(3):273–86. doi: 10.1002/jcb.240550303 7962158 10.1002/jcb.240550303

[57] Mueller M, Schilling T, Minne HW, Ziegler R. A Systemic Acceleratory Phenomenon (Sap) Accompanies the Regional Acceleratory Phenomenon (Rap) during Healing of a Bone Defect in the Rat. J Bone Miner Res. 1991;6(4):401–10. doi: 10.1002/jbmr.5650060412 1858523 10.1002/jbmr.5650060412

[58] Funk JF, Krummrey G, Perka C, Raschke MJ, Bail HJ. Distraction Osteogenesis Enhances Remodeling of Remote Bones of the Skeleton: A Pilot Study. Clin Orthop Rel Res. 2009;467(12):3199–205. doi: 10.1007/s11999-009-0902-y 19475465 10.1007/s11999-009-0902-y PMC2772934

[59] Lambers FM. Functional bone imaging in an in vivo mouse model of bone adaptation, aging and disease: Zürich: ETH; 2011.

[60] Chambers TJ, Evans M, Gardner TN, Turner-Smith A, Chow JW. Induction of bone formation in rat tail vertebrae by mechanical loading. Bone Miner. 1993;20(2):167–78. . 8453332 10.1016/s0169-6009(08)80025-6

[61] Chow JWM, Jagger CJ, Chambers TJ. Characterization of Osteogenic Response to Mechanical Stimulation in Cancellous Bone of Rat Caudal Vertebrae. Am J Physiol. 1993;265(2):E340–E7. 8368304 10.1152/ajpendo.1993.265.2.E340

